# Practical Departments

**Published:** 1904-08-13

**Authors:** 


### PRACTICAL DEPARTMENTS.

### STOVES.

In reference to a notice of their stoves, Messrs. Barnard, Bishop and Barnards, of Norwich, state that the register door and rack are quite loose and are always removed bodily from the frame when the chimney is swept.

